# Genomic Heterogeneity of Breast Tumor Pathogenesis

**DOI:** 10.4137/cmo.s2946

**Published:** 2009-07-29

**Authors:** Rachel E. Ellsworth, Jeffrey A. Hooke, Craig D. Shriver, Darrell L. Ellsworth

**Affiliations:** 1 Clinical Breast Care Project, Henry M. Jackson Foundation for the Advancement of Military Medicine, 620 Seventh Street, Windber, PA, 15963, USA.; 2 Clinical Breast Care Project, Walter Reed Army Medical Center, 6900 Georgia Avenue, NW, Washington, DC, 20307, USA.; 3 Clinical Breast Care Project, Windber Research Institute, 620 Seventh Street, Windber, PA, 15963, USA.

**Keywords:** breast cancer, tumor grade, genetic pathways

## Abstract

Pathological grade is a useful prognostic factor for stratifying breast cancer patients into favorable (low-grade, well-differentiated tumors) and less favorable (high-grade, poorly-differentiated tumors) outcome groups. Under the current system of tumor grading, however, a large proportion of tumors are characterized as intermediate-grade, making determination of optimal treatments difficult. In an effort to increase objectivity in the pathological assessment of tumor grade, differences in chromosomal alterations and gene expression patterns have been characterized in low-grade, intermediate-grade, and high-grade disease. In this review, we outline molecular data supporting a linear model of progression from low-grade to high-grade carcinomas, as well as contradicting genetic data suggesting that low-grade and high-grade tumors develop independently. While debate regarding specific pathways of development continues, molecular data suggest that intermediate-grade tumors do not comprise an independent disease subtype, but represent clinical and molecular hybrids between low-grade and high-grade tumors. Finally, we discuss the clinical implications associated with different pathways of development, including a new clinical test to assign grade and guide treatment options.

## Introduction

Breast cancer is the most common cancer in women worldwide, and in the United States (US) is estimated to account for ~26% of all new female cancer cases and 15% of all cancer deaths among women.^1^ Incidence of breast cancer in the US has risen by approximately 1.2% per year since 1930,^2^ such that one in eight American women now are expected to develop breast cancer during her lifetime. Research attempting to understand the molecular nature of breast cancer and its progression will have a tremendous impact on costs associated with disease, and importantly, on the nature of diagnosis, treatment, and prevention.

Pathological assessment of breast cancer is currently based on criteria such as tumor size, lymph node and hormone receptor status, and epidermal growth factor receptor 2 (HER2) expression, but pathology alone does not accurately predict outcomes, even for patients with similar tumor characteristics. Recent studies suggest that, despite use of identical treatment modalities in patients with similar pathological characteristics, clinical outcomes can be highly variable.^3^ Differences in response to treatments such as Tamoxifen and Herceptin^®^ likely reflect heterogeneity in pathological factors such as estrogen receptor (ER) and HER2 status. Breast carcinomas are heterogeneous at the molecular level, with at least five disease categories identified through differential patterns of gene expression.^4^–^6^ This extensive clinical, pathological, and molecular heterogeneity complicates diagnosis, prognosis, and treatment of patients with breast cancer.

In this review, we examine use of the Nottingham histological score in assigning grade to breast carcinomas and the clinical utility of the Nottingham score in determining patient risk and outcome. We outline our understanding of how genomic alterations contribute to histological characteristics that define tumor grade and the importance of molecular changes in shaping tumor growth and differentiation in patients with breast cancer. An important focus of this review is the ongoing debate over development of high-grade and low-grade breast disease, specifically on whether low-grade and high-grade breast carcinomas represent separate and distinct diseases. We present molecular evidence supporting a linear model of progression from low-grade to high-grade carcinomas, as well as contradicting genetic data suggesting that low-grade and high-grade tumors develop independently. While debate regarding specific pathways of development continues, molecular data suggest that intermediate-grade tumors do not comprise an independent disease subtype, but represent clinical and molecular hybrids between low-grade and high-grade tumors. Finally, we discuss the clinical implications of different pathways of development, including a new clinical test to assign grade and guide treatment options.

## Nottingham Histological Score

The Nottingham combined histological grading system, based on classification parameters developed by Bloom and Richardson^7^ as modified by Elston and Ellis,^8^ is currently the most widely used method for assessing breast tumor grade. The Nottingham score uses three components, tubule formation, nuclear pleomorphism, and mitotic count, each of which are scored independently using criteria described in Table 1.^9^ Scores for the three components are then combined and the cumulative score serves as the classifier: low-grade (well-differentiated) tumors have a cumulative score of 3, 4, or 5; intermediate-grade (moderately-differentiated) tumors score 6 or 7; and high-grade (poorly-differentiated) tumors have cumulative scores of 8 or 9.

The Nottingham grading system has clinical utility in determining patient risk and outcome—patients with low-grade carcinomas have ~95% five-year survival compared to just 50% in patients with high-grade disease.^8^,^10^ Although the prognostic power of the Nottingham score has prompted the College of American Pathologists to suggest using grade during staging,^11^ grade has not yet been incorporated as a component of tumor staging.^12^ Use of grade is impaired by 1) the inherent subjectivity associated with its assessment—concordance between pathologists ranges from 50%–85%,^13^ and 2) the large number (30%–60%) of tumors classified as intermediate-grade (moderately-differentiated). These tumors have features of both low-grade and high-grade tumors, making it difficult to assess risk and determine the most appropriate treatment option for patients.^14^

## Genomic Discrimination of Low- and High-Grade Breast Carcinomas

Breast cancer progression can be defined by a non-obligatory sequence of histological changes from normal epithelium through atypical hyperplasia, *in situ* carcinoma, and finally invasive malignancy.^15^ The hypothesis of dedifferentiation posits that breast cancers evolve from well-differentiated to poorly-differentiated tumors following a linear model. The progressive sequence of dedifferentiation is: well-differentiated (grade 1) → moderately-differentiated (grade 2) → poorly-differentiated (grade 3). Support for a link between histological progression and tumor growth comes mainly from clinical studies, which have identified correlations between histological grade and tumor size,^16^ or observed that impalpable carcinomas detected by mammography tend to be well-differentiated.^17^ In contrast, observations that recurrent carcinomas tend to exhibit the same level of cellular differentiation, and hence the same histological grade, as the original primary tumor^18^ have led to the hypothesis that low-grade and high-grade carcinomas reflect different disease entities. Although certain DNA copy number changes defined by comparative genomic hybridization (CGH) correlate with the degree of histological differentiation,^19^ several molecular studies suggest that the majority of low-grade (well-differentiated) tumors do not progress to high-grade (poorly-differentiated) carcinomas. For example, Roylance et al^20^ observed distinct genomic differences between grade I and grade III breast tumors; in particular, loss of chromosome 16q was significantly more frequent in grade I (65%) compared to grade III (16%) tumors. Likewise, Buerger et al^21^ observed frequent loss of chromosome 16q in well-differentiated invasive breast carcinomas and concluded that sequential progression from low-grade to high-grade is unlikely because chromosomal alterations at 16q were not maintained in higher-grade tumors.

**Figure 1.**
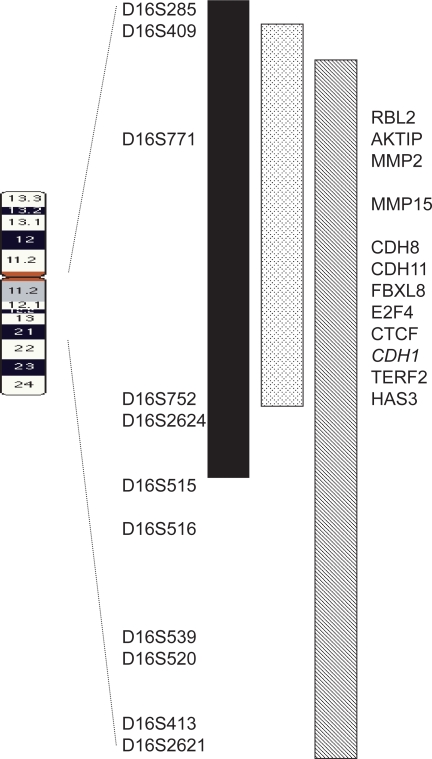
Map of common region of LOH/AI on chromosome 16q in low-grade breast carcinomas. Slight variations in the boundaries of the region have been reported: black bar, (20); checked bar, (23); striped bar, (24). Candidate genes located in the region are shown on the right. Note that mutations in CDH1 have been associated with invasive lobular carcinoma, but not with low-grade or high-grade invasive ductal carcinoma. **Abbreviations:** RBL2, retinoblastoma-like 2; AKTIP, akt-interacting protein; MMP, matrix metalloproteinase; CDH, cadherin; FBXL8, f-box and leucine-rich repeat protein 8; E2F4, e2f transcription factor 4; CTCF, CCCTC-binding factor; TERF2, telomeric repeat-binding factor 2; HAS3, hyaluronan synthase 3.

Since the initial models of disease progression were published, a number of studies examining levels and patterns of genomic variation in breast carcinomas have supported the hypothesis that low-grade and high-grade tumors represent separate genetic diseases, based largely on observations that the frequency of alterations at chromosome 16q was significantly higher in low-grade tumors. An allelic imbalance (AI) analysis using three microsatellite markers on chromosome 16q detected a significantly higher frequency of AI events in low-grade (grade 1) compared to high-grade (grade 3) tumors for two of the three markers.^22^ Likewise, microsatellite-based data from our own group showed significantly higher levels of AI at chromosome 16q11-q22 in low-grade compared to high-grade breast carcinomas.^23^ In addition, low-grade tumors contained larger alterations across the 23 Mb region of chromosome 16 compared to high-grade tumors. Only proximal markers (D16S409 and D16S2624) on 16q had a higher frequency of AI in grade 1 versus grade 3 tumors, suggesting that changes in the 16q11-q22 region are critical in the development of low-grade disease (Fig. 1). Similarly, an assessment of copy number status across chromosome 16q by CGH, AI, and fluorescence *in situ* hybridization (FISH) demonstrated that low-grade and high-grade disease were associated with different types of chromosomal alterations in the 16q region.^24^ Physical loss of large portions of chromosome 16q was associated with low-grade disease, while small regions of loss of heterozygosity (LOH) were characteristic of high-grade tumors. Further, the timing of alterations at 16q appeared to differ between tumor grades, with physical loss of 16q being an early and critical event in the development of low-grade breast tumors, while smaller alterations of 16q occurred late in the development of high-grade carcinomas.^24^ Together, these studies support a model by which low-grade and high-grade diseases develop along separate genetic pathways, with alterations of chromosome 16q serving as the critical genetic determinant between histological grades.

**Figure 2.**
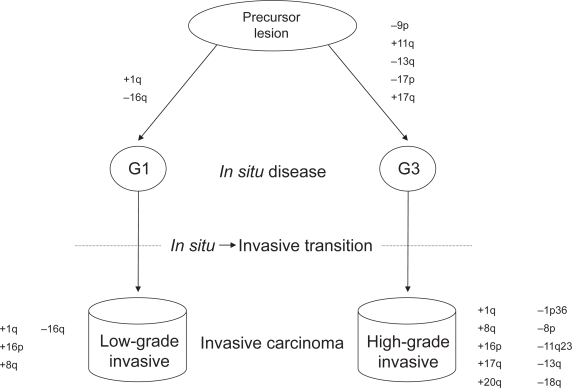
Models of genomic changes depicting pathways of development for low-grade and high-grade breast carcinomas. Low-grade DCIS and IDCA are genetically distinct from high-grade *in situ* and invasive disease. Alterations of chromosomes 1q, 8q, 16p, and 16q are associated with low-grade disease, while high-grade tumors demonstrate higher levels of alterations at a number of regions throughout the genome. **Abbreviations:** G1, grade 1; G3, grade 3; IDCA, invasive ductal carcinoma.

Molecular characterization of ductal carcinoma *in situ* (DCIS) lesions further supports a model of two distinct pathways of breast disease development. Higher levels of chromosomal alterations have been detected via CGH in high-grade compared to low-grade DCIS, with loss of 16q found almost exclusively in low-grade lesions.^25^,^26^ AI analysis on 100 pure DCIS specimens (with no detectable invasive component) recently found significantly higher levels of AI in the high-grade compared to low-grade lesions—AI at chromosome 16q characterized low-grade lesions, while alterations at 6q25–q27, 8q24, 9p21, 13q14, and 17p13.1 were frequent in high-grade disease.^27^ Similar patterns of chromosomal changes in *in situ* and invasive disease suggest that low-grade and high-grade invasive breast tumors evolve directly from low-grade and high-grade DCIS, respectively (Fig. 2).

### Components of the Nottingham score

The Nottingham score uses three components, tubule formation, nuclear pleomorphism, and mitotic count, to assign histological grade, but mechanisms by which genomic changes in breast carcinomas specifically contribute to these underlying components are unknown. When patterns of AI were compared between tumors with favorable (= 1) and unfavorable (= 3) scores for each component, significantly higher levels of AI were observed in samples with unfavorable (high) scores for all components.^28^ Tumors with reduced tubule formation (score = 3) showed higher levels of AI at chromosomal regions 11q23 and 13q12, those with high levels of nuclear atypia had frequent alterations at 9p21, 11q23, 13q14, 17p13, and 17q12, and carcinomas with high mitotic counts were commonly altered at 1p36, 11q23, and 13q14. Only region 16q11–q22 was altered more frequently in samples with low nuclear atypia. Alterations at 11q23 are common in breast tumors showing reduced tubule formation, high nuclear atypia, and high mitotic counts, suggesting that this is an early genetic change in the development of poorly-differentiated breast tumors; however, alterations at other chromosomal regions in poorly-differentiated tumors may specifically influence cell structure, nuclear morphology, and cellular proliferation.

### Genomic heterogeneity and breast cancer

The identification of genomic signatures for low-grade and high-grade breast disease provides new insights into the heterogeneity of breast cancer. Under current models of disease progression, low-grade and high-grade breast carcinomas develop independently along different genetic pathways, thus consideration of breast disease without regard to tumor grade may mask molecular (or environmental) factors specific to one grade.^20^ For example, grade 1 DCIS has been shown to exhibit a significantly lower overall frequency of chromosomal changes than low-grade (well-differentiated) invasive carcinomas, but no individual chromosomal regions effectively differentiate low-grade *in situ* from invasive disease. In contrast, high-grade (poorly-differentiated) invasive tumors did not show significantly higher levels of AI than grade 3 DCIS, but AI events at specific chromosomal regions (1p36 and 11q23) were significantly more frequent in high-grade invasive tumors compared to high-grade DCIS.^29^ Lower levels of AI in low-grade *in situ* lesions compared to low-grade invasive carcinomas may reflect the protracted time-to-progression associated with low-grade DCIS. Likewise, increased levels of AI at 1p36 and 11q23 in high-grade carcinomas suggest that these chromosomal regions may harbor genes associated with invasiveness. Therefore, consideration of histological grade when analyzing genetic data has the potential to identify molecular changes associated with invasion and to define molecular signatures of aggressive behavior for low-grade and high-grade disease.

## Molecular Evidence for a Biological Continuum

Stratification of low-grade and high-grade breast carcinomas into separate molecular diseases is based on the high frequency of alterations observed for chromosome 16 in low-grade tumors and a low frequency of 16q alterations in high-grade tumors. To localize genes involved in low-grade IDCA, and to refine regions of chromosome 16 that may be important to the development of high-grade disease, Roylance et al characterized 40 low-grade (grade 1) and 17 high-grade (grade 3) IDCA using CGH with nearly contiguous coverage of chromosome 16q.^30^ The majority of low-grade tumors showed large deletions of 16q, while high-grade tumors were more frequently characterized by multiple, small chromosomal alterations, including copy number gains in this region. Because many regions demonstrated loss and gain of certain sections, chromosome 16q may be inherently unstable and many of these regions may contain secondary, rather than causative, alterations. Based on this data, and the identification of copy number gains not previously detected, Roylance et al^30^ suggest that loss of chromosome 16q is an early event in the development of low-grade tumors, and postulate that high-grade carcinomas evolve from low-grade tumors by the accumulation of subsequent chromosomal alterations, such as small breaks and amplifications. These observations question the role of chromosome 16q deletions as the key to defining low- and high-grade genetic pathways of development.

The Nottingham grading system, used to assign histological grade to invasive carcinomas, does not adequately describe internal variation in the degree of differentiation within tumors. Although most pathologists rely on nuclear grade, either alone or in combination with central necrosis, to classify DCIS, one recent attempt to quantify histological diversity in 120 pure DCIS lesions found that ~46% of cases showed localized variability in histological grade. Nearly one-third of lesions with internal grade differences demonstrated further diversity for a panel of immunohistochemistry markers including ER, GATA-binding protein 3 (GATA3), and HER2.^31^ The authors concluded that higher-grade DCIS gradually evolve from lower-grade *in situ* lesions by random accumulation of genetic mutations. These studies hypothesize that low-grade and high-grade breast carcinomas are not necessarily unique genetic diseases. Under this model, cells with the most aggressive/poorly-differentiated characteristics tend to become the dominant cell type during progression from low-grade to high-grade carcinomas.

## Molecular Classification of Intermediate-Grade Tumors

Molecular and pathological changes have been associated with low-grade and high-grade breast carcinomas, which represent the extremes of histological differentiation. Conversely, development of a model for intermediate-grade breast tumors presents a particular challenge because intermediate-grade carcinomas contain a blend of histological features common to both low-grade (well-differentiated) and high-grade (poorly-differentiated) tumors. Because carcinomas with intermediate-grade histology represent 30%–60% of all invasive breast cancers, improved understanding of the genetics of these tumors is critical in determining the optimum course of treatment for the large group of patients with intermediate pathological features.^32^

Patterns of genetic changes usually do not differ significantly between intermediate-grade and high-grade carcinomas, supporting the idea that intermediate-grade invasive breast tumors develop from either grade 2 or grade 3 DCIS. Further studies of genomic alterations in breast tumors of different histological grades have shown that although genetic changes were more frequent in grade 3 tumors, alterations of one specific chromosomal region (16q) were significantly lower (P < 0.01) in high-grade (26%) compared to intermediate-grade (54%) tumors.^33^ Thus it appears that intermediate-grade carcinomas may represent a mixture of histological characteristics and may develop along two independent genetic pathways, one characterized by loss of chromosome 16q, few genomic alterations, and high rates of diploidy, while the other pathway is characterized by high homology with high-grade tumors.

In our ongoing studies of intermediate-grade breast carcinomas, we observed that clinicopathological characteristics and overall levels of genomic alterations in grade 2 tumors were generally intermediate compared to low-grade and high-grade disease.^23^ Specifically, 47% of the intermediate-grade tumors showed patterns of genomic alterations similar to high-grade tumors, while 11% had a low-grade signature where AI was detected only at chromosome 16q. Of note, 24% of cases showed genetic features representing a mixture of low-grade and high-grade disease, while 18% had a unique genomic profile not observed in either high- or low-grade tumors. These data suggest that intermediate-grade carcinomas should not be classified as a discrete disease type, but represent a blend of low-grade and high-grade diseases.

Gene expression analysis has been widely used to identify genetic profiles associated with different stages of breast cancer development. Using laser microdissection to isolate pure populations of tumor cells and prevent cross-contamination from stroma or co-occurring lesions, histological grade rather than pathological stage, was found to correlate with significantly different patterns of gene expression.^34^ A subset of samples showed gene expression signatures that were distinctly grade-1-like or grade-3-like; most intermediate-grade tumors exhibited a mixed low- and high-grade gene expression profile. Similarly, an expression signature from only five genes—barren homologue—Drosophila (BRRN1), hypothetical protein FLJ11029, chromosome 6 open reading frame 173 (C6orf173), serine/threonine-protein kinase 6 (STK6), and maternal embryonic leucine zipper kinase (MELK)—has been shown to discriminate low-grade from high-grade tumors with ~95% accuracy.^35^ When applied to intermediate-grade tumors, ~66% (83/126) were reclassified as low-grade-like (G2a) and 34% (43/126) as high-grade-like (G2b). Only five samples had true intermediate gene expression scores. Survival outcomes for the G2a and G2b groups were similar to those in patients with grade 1 and grade 3 tumors, respectively. Further investigation of intermediate-grade carcinomas classified as G2a and G2b demonstrated marked heterogeneity between the two groups, suggesting that intermediate grade tumors do not represent an independent disease subtype and that the G2a and G2b classifications should be considered separate pathobiological entities.^35^

Recognizing the inherent subjectivity in assigning histological grade and the need to better characterize intermediate-grade tumors, researchers have begun to analyze combined gene expression data sets from primary breast tumor samples derived from multiple sources. These approaches have led to the development of a gene expression grade index (GGI), based on 97 genes, which summarizes molecular differences between low-grade and high-grade breast tumors.^14^ Similar to earlier results,^34^ the GGI partitions intermediate-grade carcinomas into low-grade and high-grade clusters; with un-clustered cases representing a mixture of the two grades.

## Clinical Implications

Molecular data (DNA and RNA) suggest that intermediate-grade invasive breast cancer is not a discrete disease, but represents a blend between low-grade and high-grade tumors. However, whether poorly-differentiated tumors arise from well-differentiated carcinomas, or whether low-grade and high-grade tumors develop along independent genetic pathways remains unclear. Although multiple studies have identified significant differences in gene expression between low-grade and high-grade disease,^14^,^34^,^35^ gene and protein expression profiles are transient, reflecting biological conditions in the tumor at the time of excision, rather than an evolutionary history of tumor development. In contrast, chromosomal changes can be very useful for modeling disease progression. Continuing improvements in technologies to measure chromosomal alterations, such as copy number changes assessed by large-scale single-nucleotide polymorphism (SNP) arrays,^36^ may provide the tools necessary to determine the role of chromosome 16q in the development of low-grade tumors and further examine the development of low-grade as well as high-grade breast carcinomas.

Determining relationships among tumors of different histological grades has important clinical implications for estimating risk and defining treatment options in patients with breast disease. For example, atypical ductal hyperplasia (ADH) specimens typically share a gene expression profile with grade 1 disease and tend to cluster with low-grade DCIS and well-differentiated invasive cancer.^34^ Thus, ADH may represent a precursor lesion, specifically to low-grade breast cancer. Should low-grade disease be genetically distinct from high-grade, patients diagnosed with ADH could be considered lower-risk, reflecting the less aggressive phenotype of low-grade disease. Similarly, under a model of tumor progression from low-grade to high-grade through histological de-differentiation, identification of molecular changes that promote progression may provide molecular targets for the development of therapeutics to block the progression from low-grade to high-grade (aggressive) tumors.

Development of molecular signatures that closely correlate with histological differentiation may improve the assessment of tumor grade. At present, debate continues within the pathology community over the best way to assign histological grade. Some studies suggest that a two-tiered grading system comprised of nuclear pleomorphism and mitotic counts is superior to the current tripartite system that includes tubule formation.^9^,^37^ In contrast, research suggests that a composite score based on a 7-point scale (range 3–9) is more accurate than the current system that converts the cumulative scores from tubule formation, nuclear pleomorphism, and mitotic counts to a 3-grade system.^38^ For example, while tumors with a composite score of 6 or 7 would be classified as intermediate-grade, those with a score of 7 have a prognosis similar to high-grade tumors. It is possible that tumors with a score of 6 correspond to the G2a tumor group and those with a score of 7 to the G2b tumor group defined by Ivshina et al^35^ suggesting that tumors with scores of 6 and 7 should be considered separately when making treatment decisions.

Finally, molecular profiles such as the Onco*type*DX™ (Genomic Health, Redwood City, CA) and MammaPrint^®^ (Agendia, Amsterdam, The Netherlands) are now being used more frequently as clinical tools to determine treatment for certain groups of patients. For example, the Onco*type*DX™ can be used to make decisions about chemotherapy after surgery for women with node-negative, ER-positive breast cancer. In 2008, Ipsogen (http://www.ipsogen.com/) developed the MapQuant Dx™ Genomic Grade test based on the GGI discussed above.^14^ This test is being marketed as the first microarray-based diagnostic test to measure tumor grade. With the reported ability to classify 80% of intermediate-grade tumors as either low-grade or high-grade, the MapQuant assay may be useful in guiding treatment options, possibly sparing patients with grade 1 or grade 1-like tumors unnecessary treatments, while identifying patients who would benefit from chemotherapy.^32^

## Summary

Molecular characterization of breast tumors at both the DNA and RNA levels suggests that intermediate-grade carcinomas do not represent an independent disease subtype, but instead share clinical and molecular features of low-grade and high-grade tumors. In contrast, debate continues as to whether poorly-differentiated (high-grade) tumors evolve from well-differentiated (low-grade) tumors or whether low-grade and high-grade carcinomas represent discrete diseases that develop along separate genetic pathways. While efforts continue to improve our understanding of biological factors influencing the development of low-, intermediate-, and high-grade tumors, clinical uses of molecular assays are providing new ways to assign histological grade and guide treatments for patients with breast cancer.

## Figures and Tables

**Table 1.** Criteria for histological grading of invasive breast carcinomas.^a^

**Component**	**Score**	**Description**
Tubule formation^b^		
	1	Majority of tumor (>75%)
	2	Moderate degree (10%–75%)
	3	Little or none (<10%)
Nuclear pleomorphism		
	1	Small, uniform nuclear size and shape
	2	Modest increase in size and variation
	3	Large with marked variation
Mitotic counts^c^		
	1	≤7
	2	8–16
	3	≥17

a Scores for the three components are combined and the cumulative score classifies breast tumors as: low-grade (well-differentiated) tumors, 3, 4, or 5; intermediate-grade (moderately-differentiated) tumors, 6 or 7; high-grade (poorly-differentiated) tumors, 8 or 9.

b Percent of carcinoma composed of tubular structures.

c Mitotic counts vary widely with microscope type. Scores provided here are per 10 high-power fields on an Olympus BX41 microscope with a field diameter 0.54 mm.
